# Lamina Cribrosa Morphology Predicts Progressive Retinal Nerve Fiber Layer Loss In Eyes with Suspected Glaucoma

**DOI:** 10.1038/s41598-017-17843-8

**Published:** 2018-01-15

**Authors:** Jeong-Ah Kim, Tae-Woo Kim, Robert N. Weinreb, Eun Ji Lee, Michaël J. A. Girard, Jean Martial Mari

**Affiliations:** 1 Department of Ophthalmology, Seoul National University College of Medicine, Seoul National University Bundang Hospital, Seongnam, 13620 Republic of Korea; 2 Shiley Eye Institute, Hamilton Glaucoma Center, Department of Ophthalmology, University of California, San Diego, 9500 Gilman Drive, La Jolla, California, 92093 USA; 3 Department of Biomedical Engineering, National University of Singapore, Singapore, 117583 Singapore; 4 Singapore Eye Research Institute, Singapore National Eye Centre, 168751 Singapore, Singapore; 5 GePaSud, University of French Polynesia, Faa’a, 98702 French Polynesia

## Abstract

Although early diagnosis and treatment reduce the risk of blindness from glaucoma, the decision on whether or not to begin treatment in patients with suspected glaucoma is often a dilemma because the majority of patients never develop definite glaucoma. A growing body of evidences suggests that posterior bowing of the lamina cribrosa (LC) is the earliest structural change preceding the retinal nerve fiber layer (RNFL) loss in glaucomatous optic neuropathy. Based on this notion, we conducted a prospective study enrolling 87 eyes suspected of having glaucoma to investigate whether the future rate of RNFL loss is associated with the baseline LC curve evaluated by measuring the LC curve index (LCCI) using enhanced depth imaging optical coherence tomography. A faster rate of RNFL loss was significantly associated with greater LCCI (*P* < 0.001;standardized coefficient beta = −0.392), older age (*P* = 0.008;beta = −0.314), and greater vertical cup-to-disc ratio (*P* = 0.040;beta = −0.233). Assessment of LC morphology may help predict the disease outcome in eyes with suspected glaucoma.

## Introduction

Glaucoma is an optic neuropathy characterized by progressive neuroretinal rim thinning, excavation, and loss of the retinal nerve fiber layer (RNFL). These structural changes are accompanied by irreversible functional loss, and structural damage often occurs earlier than a detectable visual field (VFD) defect^1^. In most studies, only a relatively small proportion of patients who were suspected of having glaucoma or who had disease-related risk factors, such as ocular hypertension, have been found to have definite glaucoma with a manifest VFD defect during follow-up^2–5^. Therefore, stratification of patients according to the likelihood of progression to glaucoma should facilitate better allocation of limited healthcare resources, allow for enhanced disease surveillance and earlier intervention for those at higher risk, and help avoid unnecessary interventions and treatment side effects in individuals deemed at low risk^6^.

Studies have shown that certain risk factors, such as older age, high intraocular pressure (IOP), thinner central cornea, disc hemorrhage (DH), and topographic optic disc measurements are predictive of the development of primary open-angle glaucoma among patients with suspected glaucoma or those with ocular hypertension^7–11^. Additionally, a visual field (VF) parameter (higher pattern standard deviation [PSD] of the Humphrey perimetry)^9,10^ and structural characteristics of the optic nerve (e.g., larger vertical and horizontal cup-to-disc [C/D] ratio or larger vertical C/D ratio asymmetry)^9–11^ have been shown to help predict eyes with a higher chance of developing definitive glaucomatous damage.

The lamina cribrosa (LC) is a mesh-like tissue composed of connective tissue, glial cells, and microvessels that support the axons of retinal ganglion cells (RGC)^12,13^. Experimental studies have shown that posterior displacement of the LC precedes early surface-detected structural damage and RNFL loss^14–17^. Leung *et al*. demonstrated that the surface of the optic nerve head becomes depressed before RNFL thinning in patients with glaucoma^18^. These findings suggest that LC deformation, such as posterior LC bowing that leads to an increase in LC curve and LC depth (LCD), may occur in the earliest stage of the disease, and that it may induce subsequent axonal damage. Based on these considerations, it has been proposed that evaluation of LC morphology is useful in predicting progression to manifest glaucoma among patients with suspected glaucoma.

The purpose of this study was to investigate whether LC morphology is predictive of the rate of RNFL thinning among patients with suspected glaucoma.

## Results

### Baseline characteristics

One hundred two eyes of 102 patients with suspected glaucoma were initially included in the study. Of these, 3 patients had received IOP-lowering treatment before 3-year follow-up and, hence, were excluded from the study. Twelve subjects were also excluded because of the insufficient visibility of the anterior LC surface, leaving a final sample of 87 subjects. The subjects’ clinical characteristics are summarized in Table 1.

**Table 1:** Demographic characteristics of the study subjects.

Variables		Glaucoma suspect (n = 87)
Age (years)		56.36 ± 10.9 (27–79)
Female gender, (%)		54 (62.1)
Diabetes mellitus, (%)		16 (18.4)
Hypertension, (%)		25 (28.7)
Family history of glaucoma (%)		7 (8.0)
Cold extremities (%)		18 (20.7)
Migraine (%)		6 (6.9)
Baseline IOP (mmHg)		13.5 ± 3.2 (7–24)
Mean IOP during follow-up (mmHg)		13.3 ± 2.5 (7.5–20.9)
IOP fluctuation during follow-up (mmHg)		1.4 ± 0.6 (0–3.8)
Diagnosis (%)		
GON (n)		96.6% (84)
OHT (n)		3.4% (3)
Refractive error (D)		−0.40 ± 1.83 (−6.13 to +2.25)
Central corneal thickness (μm)		553.9 ± 37.8 (472–645)
Axial length (mm)		23.84 ± 1.07 (21.15–26.5)
Cup to disc ratio	Horizontal	0.62 ± 0.07 (0.5–0.8)
	Vertical	0.60 ± 0.07 (0.4–0.8)
Global RNFL thickness (μm)		97.6 ± 9.4
Visual field MD (dB)		−0.13 ± 1.40 (−1.69 to 2.41)
Visual field PSD (dB)		1.65 ± 0.40 (1.09–3.47)
Average LCD		485.9 ± 107.3 (242–709)
Average LCCI		7.93 ± 1.58 (4.94–13.20)
Disc hemorrhage during follow-up, (*n*)		8.1% (7)
Follow-up period (years)		4.8 ± 1.1 (3–6)
Number of SD-OCT RNFL scans		6.7 ± 2.0 (5–13)

IOP = intraocular pressure; GON = glaucomatous optic neuropathy; OHT = ocular hypertension; D = diopters; MD = mean deviation; PSD = pattern standard deviation; LCD = lamina cribrosa depth; LCCI = lamina cribrosa curvature index; RNFL = retinal nerve fiber layer; SD-OCT = spectral-domain optical coherence tomography. Data are mean ± standard deviation or *n* (%) values.

The patients were 56.6 ± 10.8 years old (range, 27–79 years) and 57 (62%) were female. Only three eyes (3.4%) among the 87 eyes were categorized as having ocular hypertension (OHT) at the baseline examination. The average number of OCT examinations per eye was 6.7 (range, 5–13) with an average follow-up time of 4.8 years. The entire cohort had a baseline IOP of 13.5 ± 3.2 mmHg (range, 7–24 mmHg), a refractive error (spherical equivalent) of −0.40 ± 1.83 diopters (range, −6.13 to + 2.25 diopters), and a VF mean deviation of −0.13 ± 1.40 dB (range, −1.69 to 2.41 dB).

**Figure 1:**
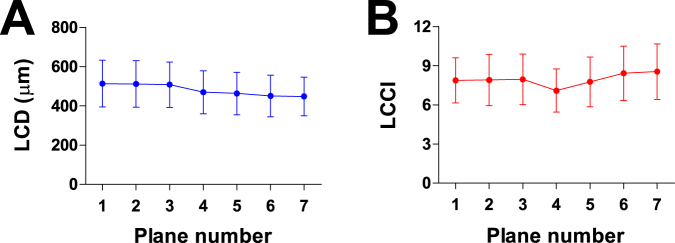
Lamina cribrosa depth (LCD, **A**) and lamina cribrosa curve index (LCCI, **B**) profiles in seven horizontal B-scan images (planes 1 and 7 correspond to the superior- and inferior-most planes, respectively) in eyes with suspected glaucoma. The LCD is largest in the superior planes and gradually decreases when moving to the inferior region. In contrast, the LCCI was greatest in inferior region followed by superior region.

The 95% Bland-Altman limits of agreement between the measurements from the two glaucoma specialists were −23.18 to 23.36 µm for the LCD and −1.22 to 1.02 for the LCCI. Average LCD and LCCI were 485.9 ± 107.3 µm (range, 242–709) and 7.93 ± 1.58 (range, 4.94–13.20), respectively. LCD was largest in the superior planes and gradually decreased when moving to the inferior region (plane 1, 2, 3 > 4 > 5, 6, 7), whereas the LCCI was largest in two most inferior planes, followed by the superior planes (plane 7, 6 > 1, 2, 3, 5 > 4) (*P* < 0.001, repeated measures ANOVA with Bonferroni’s post hoc test) (Fig. 1).

During follow-up, 6 DHs were detected in six patients (6.90%). Of these, 2 DHs were located in the superotemporal sector of the optic disc and the others were located in the inferotemporal sector. Of the 87 patients included in this study, 15 patients (17.24%) showed significant glaucoma progression and began to receive IOP-lowering treatment at 4.6 ± 1.3 years (range, 3 to 6 years) after baseline examination.

### Rate of RNFL loss

**Figure 2:**
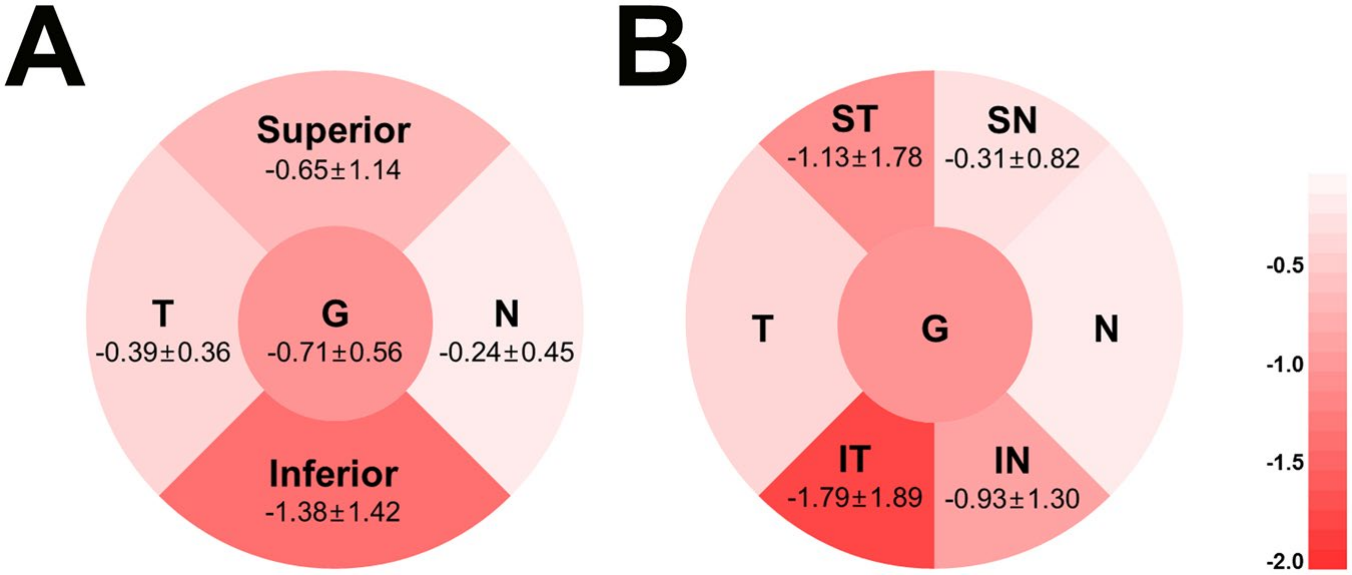
Venn diagrams showing the rates of sectoral retinal nerve fiber layer thickness change over time (µm/year) in eyes with suspected glaucoma. The rate was fastest at the inferotemporal sector. G = global; IN = inferonasal; IT = inferotemporal; N = nasal; SN = superonasal; ST = superotemporal; T = temporal.

Figure 2 shows the mean rates of global and sectoral RNFL thickness loss over time. The rates of RNFL loss were −0.71 ± 0.56 μm/year in global (range, −2.29 to 0.20 μm/year), −1.38 ± 1.42 μm/year in the inferior quadrant (range, −6.64 to 0.83 μm/year), and −0.65 ± 1.14 μm/year in the superior quadrant (range, −5.78 to 0.94 μm/year). Within-subject regional comparison of the rate of RNFL loss among the six sectors revealed that the rate was fastest in the inferotemporal sector followed by the superotemporal sector (−1.79 ± 1.89 and −1.13 ± 1.78 μm/year, respectively, *P* < 0.001). A significant inter-sectoral spatial correlation was found between the two sectors in the same horizontal planes; the rate of RNFL loss in the inferotemporal sector was significantly correlated with that in the inferonasal (*r* = 0.339, *P* = 0.001), but not with those in the inferonasal and inferotemporal sectors (*r* = 0.034, *P* = 0.752 and *r* = 0.067, *P* = 0.535, respectively). Likewise, the rate of RNFL loss in the superotemporal sector was significantly correlated with that in the superonasal sector (*r* = 0.254, *P* = 0.018), which had the same horizontal plane, but not with those in the inferonasal and inferotemporal sectors (*r* = 0.034, *P* = 0.752 and *r* = 0.067, *P* = 0.535, respectively).

### Factors associated with the rate of RNFL loss

**Figure 3:**
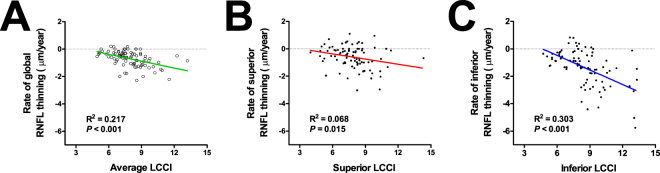
Scatterplots showing the relationship between the average (**A**) and superior (**B**) and inferior (**C**) lamina cribrosa curve index (LCCI) and the rate of retinal nerve fiber layer (RNFL) loss (µm/year). Significant relationships were observed both globally (**A**) and regionally (**B**,**C**) in the superior (**B**) and inferior (**C**) sectors.

Factors associated with the rate of RNFL loss were determined using linear regression analysis. In the univariate analysis, older age (*P* = 0.038), higher baseline IOP (*P* = 0.045), greater vertical cup-to-disc (CD) ratio (*P* = 0.002), and larger average LCD (*P* = 0.004), and greater average LCCI (*P* < 0.001) were associated with a faster global RNFL loss. Multivariate analysis revealed that older age (*P* = 0.001, standardized coefficient beta = −0.314), greater vertical CD ratio (*P* = 0.013, beta = −0.233), and greater average LCCI (*P* < 0.001, beta = −0.392) were significant factors affecting the rate of global RNFL loss (Table 2, Fig. 3A). Although significant correlations were found between some variables (supplementary Table S1), the variance inflation factors (VIFs) for the variables to be included in the univariate analysis were less than 1.5 (data not shown).

**Table 2:** Factors associated with the rate of retinal nerve fiber layer thinning (*n* = 87).

**Variables**		**Global**				**Superior sector**				**Inferior sector**			
		**Univariate**		**Multivariate**		**Univariate**		**Multivariate**		**Univariate**		**Multivariate**	
		**ß**	***P***	**ß**	***P***	**ß**	***P***	**ß**	***P***	**ß**	***P***	**ß**	***P***
**Age, per 1-year older**		**−0.012**	**0.038**	**−0.016**	**0.001**	−0.012	0.263			−0.029	0.023	−0.028	0.008
**Gender, female**		0.141	0.26			**0.586**	**0.016**	**0.518**	**0.042**	**−0.163**	**0.574**		
Diabetes mellitus		−0.26	0.096	−0.07	0.445	−0.195	0.529			**−0.882**	**0.013**	−0.104	0.902
Hypertension		0.064	0.637			−0.036	0.892			0.236	0.447		
Family history of glaucoma		0.32	0.151			0.397	0.369			**0.916**	**0.074**	0.02	0.88
Cold extremities		0.017	0.913			0.036	0.903			0.384	0.268		
Migraine		0.181	0.452			0.151	0.751			0.837	0.129		
Baseline IOP, mmHg		**−0.039**	**0.045**	−0.32	0.052	−0.052	0.173			−0.073	0.1		
Mean IOP during follow-up, mmHg		−0.031	0.202			−0.05	0.301			−0.047	0.397		
**IOP fluctuation during follow-up, mmHg**		−0.140	0.140			−0.06	0.752			−0.285	0.195		
Refractive error (spherical equivalent), diopters		0.024	0.482			0.102	0.122			0.005	0.95		
Central corneal thickness, μm		−0.001	0.777			**−0.006**	**0.08**	−0.13	0.228	0.001	0.835		
Axial length, mm		−0.02	0.749			−0.088	0.501			0.092	0.53		
Visual field MD, dB		−0.023	0.691			−0.026	0.805			0.033	0.795		
Visual field PSD, 1dB		0.168	0.403			0.191	0.640			0.279	0.526		
**Cup to disc ratio**	Horizontal	**−1.486**	**0.074**	0.120	0.367	**−3.059**	**0.062**	−0.141	0.187	**−0.345**	**0.071**	−0.111	0.449
	**Vertical**	**−2.651**	**0.002**	**−1.890**	**0.013**	**−4.174**	**0.015**	−0.144	0.186	**−4.385**	**0.029**	**−3.274**	**0.040**
Baseline RNFL thickness, μm*		0.006	0.354			−0.002	0.742			−0.007	0.421		
Number of SD-OCT scan		−0.010	0.761			0.083	0.180			**−0.122**	**0.087**	−0.084	0.952
LCD^†^	Average of 7 planes	**−0.002**	**0.004**	−0.033	0.797								
	Average of superior- most 2 planes					−0.002	0.081	0.068	0.597				
	Average of inferior- most 2 planes									**−0.004**	**0.003**	−0.025	0.530
LCCI^†^	Average of 7 planes	**−0.167**	**<0.001**	**−0.14**	**<0.001**								
	Average of superior- most 2 planes					**−0.218**	**0.001**	**−0.192**	**0.005**				
	Average of inferior- most 2 planes									**−0.354**	**<0.001**	**−0.328**	**< 0.001**

IOP = intraocular pressure; MD = mean deviation; PSD = pattern standard deviation; RNFL = retinal nerve fiber layer; LCD = lamina cribrosa depth; LCCI = lamina cribrosa curve index. *Baseline RNFL thickness means circumpapillary RNFL thickness at corresponding area. ^†^The average values were defined as the mean values of the measurements from the 7 planes (from superior to inferior peripheral plane). Only variables with *P* < 0.1 on univariate analysis were included in the multivariate model. Values with statistical significance are in boldface.

A sub-analysis was performed to determine factors associated with the rate of RNFL loss rate in each of the superior and inferior sectors. Male gender (*P* = 0.016), greater vertical CD ratio (*P* = 0.015), and greater average LCCI of the superior-most two planes (*P* < 0.001) were associated with faster RNFL loss in the superior sector in univariate analysis. Of these factors, male gender (*P* = 0.042, beta = −0.220) and greater average LCCI of the superior-most two planes (*P* = 0.005, beta = −0.303) remained significantly associated with faster RNFL loss in the superior sector. In the inferior sector, older age (*P* = 0.023), presence of diabetes (*P* = 0.013), greater vertical CD ratio (*P* = 0.029), larger average LCD of the inferior-most two planes (*P* = 0.003), and greater average LCCI of the inferior-most two planes (*P* < 0.001) were associated with faster RNFL loss in the univariate analysis. Among these factors, older age (*P* = 0.008, beta = −0.239), greater vertical CD ratio (*P* = 0.040, beta = −0.184), and greater average LCCI of the inferior-most two planes (*P* < 0.001, beta = −0.512) remained significantly associated with faster RNFL loss in the multivariate analysis (Table 2, Fig. 3A,B).

### Spatial correlation between LCCI and rate of RNFL loss

In the within-subject regional comparison (i.e., intra-eye comparison between the superior and inferior sectors), large variation was observed in terms of differences in regional LCCI difference. The difference in the LCCI between the average superior- and inferior-most two planes (i.e., the average LCCI of inferior-most two planes minus that of superior-most two planes) ranged from +6.52 to −5.47. In 56 (64.4%) subjects, the LCCI was greatest in the inferior-most two planes. In the remaining 31 (35.6%) patients, LCCI was greatest in the superior-most two planes. None of the subjects had the greatest LCCI in the mid-horizontal plane.

The sectoral rate of RNFL loss significantly corresponded with the LCCI at the corresponding region, i.e. the rate of RNFL loss was faster in the superior sector than in the inferior sector in eyes with a greater LCCI in the superior sector and vice versa (*P* = 0.006, Pearson’s chi-square test).

### Spatial correspondence between LCCI and DH

Of the 6 patients who had DH, 2 had DH in the superotemporal sector and the remaining 4 in the inferotemporal sector. The inferior LCCI was larger than the superior LCCI in the 4 patients with inferotemporal DH. The 2 patients with superotemporal DH had larger LCCI superiorly.

### Representative cases

**Figure 4:**
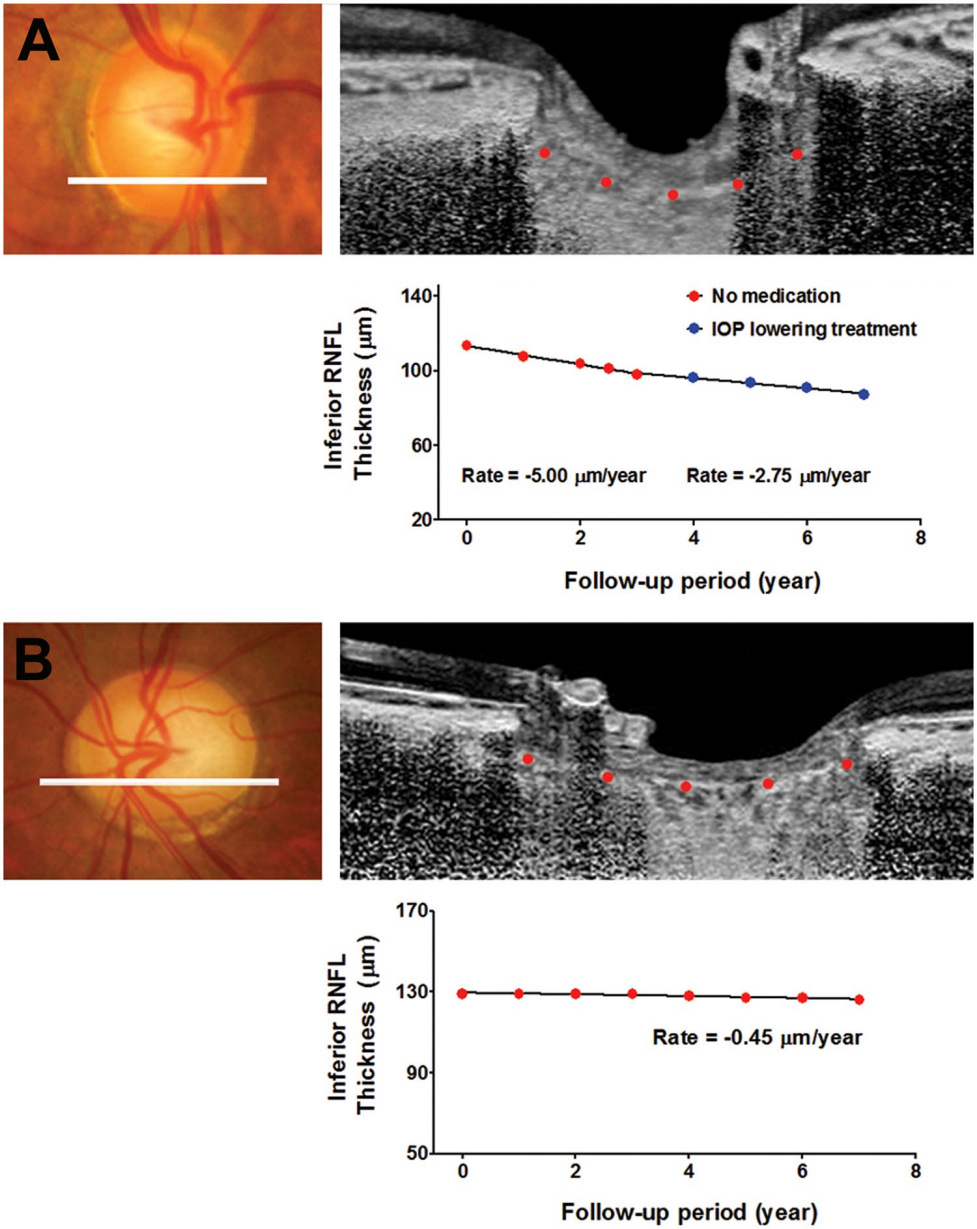
Representative cases. (**A**) A glaucoma suspect eye of a 61-year-old female with steeply curved lamina cribrosa (LC). The untreated intraocular pressure (IOP) and central corneal thickness were 16 mmHg and 537 µm, respectively. During the first 3 years following baseline examination, progressive retinal nerve fiber layer (RNFL) loss was observed in the inferior sector with the rate of −5.00 µm/year. After IOP-lowering treatment, the rate of RNFL loss decreased to −2.75 µm/year. (**B**) A glaucoma-suspected eye of a 74-year-old female with a relatively flat LC. The untreated IOP and central corneal thickness were 16 mmHg and 545 µm, respectively. During seven years of follow-up, the rate of RNFL loss in the inferior sector was −0.45 µm/year.

Two representative cases showing the association between the LCCI and the rate of RNFL loss are presented in Fig. 4.

## Discussion

Although lowering IOP treatment reduces the risk of glaucoma development in eyes with suspected glaucoma, only a small proportion of patients with suspected glaucoma progress to definite glaucoma when left untreated^3,19,20^. Moreover, treatment-related side effects reduce the quality of life of patients^21^. Thus, the decision to initiate lowering IOP treatment in patients with suspected glaucoma should be based on the patient’s risk for developing visual loss to avoid unnecessary treatment. The present study found that the eyes with steeply curved LC lost RNFL more rapidly compared with eyes with a flat LC measurement among eyes with suspected glaucoma. Considering that eyes with a faster rate of RNFL loss have a higher risk of developing VF loss in patients with suspected glaucoma^22^, the notion that steepness of the LC curve is associated with future rate of RNFL loss is clinically meaningful in regards of efficient prevention of glaucomatous VF loss.

Previous studies have demonstrated that LC deformation, which precedes the RNFL loss^14,23,24^, occurs as a posterior bowing^15,17^, Conversely, the LC curve becomes less curved after trabeculectomy in primary-open-angle glaucoma (POAG) patients^25^. Moreover, Lee *et al*. reported that LCCI had a diagnostic capability comparable to RNFL thickness measurements to discriminate eyes with glaucoma from healthy eyes^26^. These findings suggest that LC bowing is a characteristic feature of glaucoma and that LCCI is a valid parameter to assess glaucoma-associated LC deformity. For this reason, we used the LC curve index to assess the LC morphology in the present study.

The LCCI was measured using horizontal B-scan images. Because there is a horizontal ridge at or near the mid-horizontal optic nerve head (ONH)^27^, the LC is generally less displaced in this region in both healthy and glaucomatous eyes^27^. This results in a W-shaped configuration of the anterior LC surface in vertical and oblique scans (i.e., the LC would have at least two separate curvatures (Supplementary Video S1). Therefore, analysis of the LC configuration using vertical or oblique scans would be complicated. In contrast, LC has a relatively regular configuration in the horizontal plane with a flat or U shaped appearance with differing regional steepness, allowing the measurement of LCCI (Supplementary Video S2).

The LCD was not associated with the rate of glaucoma progression in the multivariate analysis. This finding is likely to be related with its limitation as a parameter to represent the LC strain. Since the LCD was measured from Bruch’s membrane opening (BMO), it should be influenced by the choroidal thickness, which is known to be variable among individuals^28^. The LC is a sieve-like perforation in the posterior part of the sclera, which is sustained by load-bearing connective tissues of the peripapillary sclera. Therefore, the choroid is not relevant to the anteroposterior LC deformation. Thus, including the choroidal thickness in the LCD would lead to a biased assessment of LC morphology^26^. Our group previously reported that there is a substantial overlap for the LCD between healthy and glaucoma patients^26^.

There was a substantial variation among individual patients in the superior-inferior regional differences in the LCCI, and the rate of RNFL loss had a significant spatial correlation with the LCCI in the corresponding area of the ONH. These results suggest that LC deformation occurs with a regional difference between the superior and inferior hemi-optic nerve head, and that glaucomatous damage first develops in the region with more severely deformed LC. The spatial correlation between the rate of RNFL loss and LCCI supports the notion that LC deformation is a key pathomechanism of glaucoma development and progression.

DH has been a known marker of rapid glaucoma progression^29,30^. However, we did not include DH as a covariate in the current study. This was because the number of eyes with DH was too small to conduct a meaningful statistical analysis. In addition, some patients who were stable during the first two years were followed up only every 12 months thereafter. In such cases, there is a substantial possibility that DH may have been missed as DH typically lasts only 6–12 weeks^31^. Therefore, dividing the patients in the present study into DH positive and DH negative groups would be problematic.

There was a spatial correspondence between LCCI and DH. This finding may support that the DH may result from the disruption of capillaries within the laminar beams^32^. However, it cannot be ruled out that the observed spatial correspondence is simply an incidental finding due to small number of patients who had DH. Further study is needed to investigate the relationship between LCCI and DH.

IOP-related parameters are important factors for glaucoma progression^33–35^. However, none of IOP-related parameters were associated with the rate of RNFL thinning in the current study. This discrepancy may be the result of a relatively low baseline and average IOP during follow-up (13.5 ± 3.1 and 13.3 ± 2.5 mmHg, respectively) together with a low proportion of OHT (3.3%) in our subjects.

In the present study, we did not find a significant association between higher baseline PSD and increased risk of the glaucoma progression in contrast to earlier studies^9,10,22^. This may be attributed to differences in the study design and participants between studies. In particular, previous studies used the conversion to POAG as an outcome while we measured the rate of RNFL loss. PSD can also be an outcome for studies evaluating the VF change. Therefore, baseline PSD is more likely to be determined as a significant predictor for disease progression in a study based on VF testing. In addition, race of study participants and proportion of OHT patients among participant in our study are largely different from those in previous studies.

Although there was a significant tendency that eyes with larger LCCI had a fast rate of RNFL loss, there were a few patients who did not progress fast despite their large LCCI. The reason underlying this observation is unclear. One possible hypothesis is that the LC in those patients may be originally steeply curved from birth. Second, those patients may have some compensatory mechanisms that cancel the effect of LC strain at least partly. Another possibility is that, if followed over a longer period of time, those patients may have faster and significant slopes.

The findings of this study should be considered in the light of some limitations. First, a LC surface reference line should be set from the LC insertion points to allow precise quantification of the LC curve. However, only the LC within the BMO width was included in measurement of the LC curve in the present study, since the LC was often not visible outside of this region. Furthermore, we previously demonstrated that the LCCI measured from the whole LC (between the LC insertions) was comparable with that measured on the LC within BMO in eyes with an LC visible up to the LC insertion^25^. Thus, we consider the assessment of the LC curve within BMO as a potential surrogate for assessment of the actual LC curve. Second, we referred to the change in the LC configuration as a change in curvature; however, the curvature is a geometric entity that refers to the inverse of the radius of the arc of a circle best fitting the portion of the curve. Thus, it should be considered that the LCCI is not a parameter corresponding to an actual LC curvature and thus, further study is needed to investigate the method of calculating the real curvature of LC. Third, eyes with a tilted or torted optic disc were excluded, and so the reported findings cannot be directly applied to eyes with such conditions. Forth, only patients who were followed-up for at least three years without IOP-lowering treatment were included. Thus, patients with rapid glaucoma progression, who needed IOP lowering treatment within three years after baseline examination, might have been excluded. However, only three patients from the original cohort were excluded due to IOP lowering treatment (data not presented). Thus, the influence of excluding those patients should be negligible. Fifth, this study only considered the LC curvature at baseline examination. Further study is needed to investigate whether LC curvature changes over time and to examine the influence of LC curvature changes on progressive RNFL loss. Lastly, all patients in this study were Korean. Therefore, the results of the present findings cannot be directly applied to other ethnic populations.

In conclusion, the steeply curved LC at baseline was associated with a faster rate of RNFL thinning in eyes with suspected glaucoma. These data suggest that assessment of LC morphology may be useful in predicting future disease progression in patients with suspected glaucoma.

## Methods

This prospective study investigated the relationship between LC morphology and the rate of RNFL loss in patients with suspected glaucoma enrolled in the Investigating Glaucoma Progression Study (IGPS)^36,37^, which is an ongoing prospective study of glaucoma patients at the Glaucoma Clinic of Seoul National University Bundang Hospital Glaucoma Clinic. Written informed consent to participate was obtained from all subjects. This study was approved by the Seoul National University Bundang Hospital Institutional Review Board and conformed to the Declaration of Helsinki.

### Study subjects

Patients enrolled in the IGPS underwent a complete ophthalmic examination including the following: measurement of best corrective visual acuity; refraction test; slit-lamp biomicroscopy; Goldmann applanation tonometry; gonioscopy; dilated stereoscopic examination of the optic disc; measurement of corneal curvature (KR-1800, Topcon, Tokyo, Japan), central corneal thickness (Orbscan II, Bausch & Lomb Surgical, Rochester, NY), and axial length (IOL Master version 5, Carl Zeiss Meditec, Dublin, CA, USA); stereo disc photography (EOS D60 digital camera, Canon, Utsunomiya-shi, Tochigi-ken, Japan); spectral-domain optical coherence tomography (SD-OCT; Spectralis OCT, Heidelberg Engineering, Heidelberg, Germany); and standard automated perimetry (Humphrey Field Analyzer II 750 and 24–2 Swedish interactive threshold algorithm; Carl Zeiss Meditec).

The IGPS excluded subjects with a history of intraocular surgery other than cataract extraction and glaucoma surgery, and intraocular disease (e.g., diabetic retinopathy or retinal vein occlusion) or a neurologic disease (e.g., stroke or brain tumor) that could cause VF loss, and best corrected visual acuities (BCVA) worse than 20/40.

All patients included in the IGPS were followed up every 3 to 6 months with regular follow-up slit-lamp examinations using a 78-diopter lens or stereo disc photography, and SD-OCT RNFL thickness measured at intervals ranging from 6 months to 1 year. The present study enrolled only patients with suspected glaucoma on both eyes, and those with one eye with suspected glaucoma and the healthy fellow eye at baseline without any history of IOP-lowering treatment. Patients were required to undergo optic disc scan using EDI SD-OCT at baseline examination, and were untreated for at least 3 years with at least five serial SD-OCT RNFL thickness measurements. Eyes with suspected glaucoma were defined as those with OHT (IOP > 21 mmHg in the presence of a healthy-appearing optic disc and normal VF), a history of elevated IOP, or the appearance potential glaucoma on the optic disc (neuroretinal rim thinning or RNFL defects on masked simultaneous stereophotograph assessments) in the presence of a normal VF at the time of SD-OCT imaging^38^. Glaucomatous appearance of the optic nerve was defined as (1) disc rim loss, notching or excavation; (2) cupping of the disc (C/D ratio > 0.6); (3) C/D ratio difference between fellow eyes > 0.2; (4) DH; or (5) diffuse or localized abnormalities of the RNFL, especially in the superior- and inferior-temporal regions. A glaucomatous VFD was defined as a defect fulfilling one or more of the following criteria: (1) outside normal limits on a glaucoma hemifield test, (2) three abnormal points with a *P* < 0.05 probability of being normal and one abnormal point with *P* < 1% by PSD, or (3) a PSD of *P* < 5% confirmed on two consecutive reliable tests (fixation loss rate of ≤20% and false-positive and false-negative error rates of ≤25%).

The exclusion criteria were eyes with a BCVA worse than 20/40, a spherical equivalent of <−6.0 D or >+3.0 D, a cylinder correction of <−3.0 D or >+3.0 D, a tilted (i.e., defined as a tilt ratio between the longest and shortest diameters of the optic disc of >1.3)^39,40^, or a torted disc (i.e., defined as a torsion angle deviation of the long axis of the optic disc from the vertical meridian of >15°)^40,41^. Eyes were also excluded when a good-quality image (i.e., quality score >15) could not be obtained in more than five sections. When the quality score did not reach 15, the image-acquisition process automatically stopped, or images of the respective sections were not obtained. Only acceptable scans with a good-quality image that allowed clear delineation of the anterior border of the peripheral LC within the BMO were included to measure the LCD and the LCCI When both eyes of a patient satisfied the inclusion criteria, one eye was randomly chosen per subject.

During the follow-up period, IOP-lowering treatment was initiated when clinically meaningful progression was observed. Progression was defined as a structural (glaucomatous change confirmed by stereo optic disc, red-free RNFL photography, and/or SD-OCT) and/or functional development of reproducible glaucomatous VFD on SAP)^42,43^. In such cases, only data obtained during the untreated period were used for the analysis.

Baseline IOP was defined as the mean of at least two measurements at the baseline examination. Mean follow-up IOP was defined by averaging the IOPs measured at every visit and IOP fluctuation was determined using the standard deviation of these values.

A DH, defined as an isolated hemorrhage seen on the optic disc tissue or in the peripapillary retinal tissue connected to the optic disc rim, was detected by slit-lamp examination using a 78-diopter lens or stereo disc photography. One of these examinations was performed at every follow-up visit.

### EDI-OCT of the optic disc and adaptive compensation to evaluate LC

The LCD and degree of LC curve (from flat to steeply curved shape) were measured using optic disc B-scan images^25^. The optic nerve image was obtained using the enhanced depth imaging (EDI) technique of the SD-OCT system, which was originally developed by Spaide *et al*.^44^. Prior to disc scan, the corneal curvature of each eye were entered into the Spectralis OCT system to avoid potential magnification errors. The optic disc was imaged through undilated pupils using a rectangle subtending 10°×15° of the optic disc. This rectangle was scanned with approximately 75 B-scan section images that were separated by 30–34 μm (the distance between the scan line was determined automatically). Approximately 42 SD-OCT frames were averaged for each section. This protocol provided the best trade-off between image quality and patient cooperation^45^.

To enhance the visibility of the anterior LC surface, all disc scan images were post-processed using adaptive compensation^46–48^, and the measurement was performed using a manual caliper tool in Amira software (version 5.2.2, Visage Imaging, Berlin, Germany) by two experienced observers (JAK and TWK) who were masked to the clinical information. The average LCD and LCCI were determined as the mean values of the measurements made at seven points of the LC. Additionally, the average LCD and LCCI in the superior- and inferior-most two planes were used for intra-eye and inter-eye comparison according to the sector because the RGC axons in the superior and inferior quadrants pass through the uppermost two planes and lowermost two planes, respectively. The mean of the measurements from each of the two observers was used for analysis.

### Measurement of LCD

**Figure 5:**
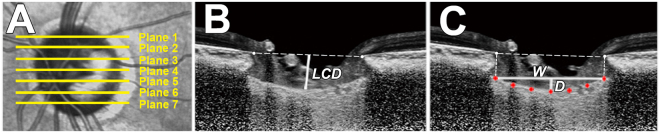
Measurement of the lamina cribosa depth (LCD) and lamina cribrosa curve index (LCCI) using B-scan images of the optic nerve head. (**A**) Infrared fundus image with lines pointing to the level of seven B-scan images spaced equidistantly across the vertical optic disc diameter in each eye. (**B**) The LCD was measured as the distance from the reference line connecting the two Bruch’s membrane opening (BMO) points to the anterior surface of the lamina cribrosa (LC). The LCD was measured at the maximally depressed point. (**C**) The LCCI was measured by dividing the LC curve depth (D) within the BMO by the width of the anterior LC surface reference line (W), and then multiplying by 100.

The LCD was measured at the seven locations equidistant across the vertical optic disc diameter using horizontal SD-OCT B-scan images. These seven B-scan lines were defined as plane 1 to plane 7 (superior to inferior regions, Fig. 5A) In this model, plane 4 corresponds to the mid-horizontal plane, and planes 2 and 6 correspond to the superior and inferior midperiphery, respectively. To determine the LCD, a line connecting the edges of BMO was set as a reference plane (BMO reference line), and then the LCD was measured in the direction perpendicular to the reference plane at the maximally depressed point (Fig. 5B).

### Measurement of LC curve

The LC curve was assessed with the LCCI using baseline disc scan images. The method to calculate LCCI has been previously described^25^. In brief, LCCI was determined by measuring the width of the LC curve reference line (LCCW) and then measuring the LC curve depth (LCCD). The LCCW was defined as the width of the line connecting the two points on the anterior LC surface that met with lines drawn from each BMO termination point perpendicular to the BMO reference line. The maximum depth from the reference line to the anterior LC surface was defined as LCCD (Fig. 5C). The LCCI was calculated as (LCCD/LCCW) × 100. Since the curvature is normalized according to LC width, it describes the shape of the LC independent of the actual size of the ONH. Only the LC within the BMO was considered because the LC was often not clearly visible outside the BMO. In eyes with LC defects, the LCD and LCCI were measured using a presumed anterior LC surface that best fit the curvature of the remaining part of the LC or excluded the area of the LC defect.

### The rate of progressive RNFL loss analysis

The RNFL thickness was measured using the parapapillary circle scan protocol of the Spectralis SD-OCT system (software version 5.4.7.0; Heidelberg Engineering, Heidelberg, Germany)^49,50^. The scan circle was centered on the center of the optic disc at baseline examination and the follow-up scan was obtained automatically by a built-in automated realignment procedure (referred to as the ‘follow-up examination’ in the system documentation). Spectralis SD-OCT images measured from April 2009 to August 2016 were included and the RNFL thickness measurements were estimated in four sectors separated into 90-degree intervals (superior, inferior, temporal, and nasal). Linear regression analysis against time was performed for the rates of global and RNFL loss in each sector for all subjects to determine the rate of RNFL loss (µm/year). The accuracy of the segmentation of the RNFL was reviewed, and segmentation errors were manually corrected by an experienced ophthalmologist (JAK) who was masked to the subjects’ clinical information.

### Statistical analysis

The inter-observer agreement for measuring the LCCI and LCD was evaluated by calculating the 95% Bland-Altman limits of agreement. A general linear model was used to investigate the factors associated with the rate of RNFL loss, first with a univariate model and then with a multivariate model that included variables from the univariate model for which *P* < 0.10. Correlations among the factors were assessed by calculating Spearman’s correlation coefficients. Collinearity between the variables was assessed by calculating the VIF. Repeated measures ANOVA was performed for within-subject regional comparison of the LCCI, LCD, and the rate of RNFL loss. The Pearson’s chi-square test was used to investigate the intra-eye influence of LCCI on the rate of RNFL loss by evaluation of the correspondence between the region with greater LCCI and the region with faster RNFL loss between the superior and inferior sectors. Probability values of *P* < 0.05 were considered statistically significant. Statistical analyses were performed using the Statistical Package for the Social Sciences software (version 22.0, SPSS, Chicago, IL, USA). The data are expressed as mean ± SD values except where stated otherwise.

### Data availability

Data supporting the findings of this study are available within the article and its supplementary information files and from the corresponding author on reasonable request.

## Electronic supplementary material

Supplementary Video legends

Supplementary Video S1

Supplementary Video S2
