# Supplementary material for: Lamina Cribrosa Morphology Predicts Progressive Retinal Nerve Fiber Layer Loss In Eyes with Suspected Glaucoma
Source: Sci Rep. 2018 Jan 15;8:738. doi: 10.1038/s41598-017-17843-8 (PMC5768684; doi:10.1038/s41598-017-17843-8)
Supplement: Supplementary file 1 — Supplementary Video legends [file 41598_2017_17843_MOESM1_ESM.pdf]

Supplementary Information

Lamina Cribrosa Morphology Predicts Progressive Retinal Nerve Fiber Layer Loss In Eyes with Suspected Glaucoma

Jeong-Ah Kim,[1] Tae-Woo Kim,[1*] Robert N. Weinreb,[2] Eun Ji Lee,[1] Michaël J. A. Girard,[3,4] Jean Martial Mari[5]

[1]Department of Ophthalmology, Seoul National University College of Medicine, Seoul National University Bundang Hospital, Seongnam, 13620, Republic of Korea

[2]Shiley Eye Institute, Hamilton Glaucoma Center, Department of Ophthalmology, University of California, San Diego, 9500 Gilman Drive, La Jolla, California 92093, USA.

[3]Department of Biomedical Engineering, National University of Singapore, 117583, Singapore

[4]Singapore Eye Research Institute, Singapore National Eye Centre, 168751, Singapore

[5]GePaSud, University of French Polynesia, Faa'a, 98702, French Polynesia

[*]Corresponding author:

Professor

Tae-Woo Kim, MD

Department of Ophthalmology, Seoul National University Bundang Hospital,

82, Gumi-ro, 173 Beon-gil, Bundang-gu, Seongnam, Gyeonggi-do 13620, Korea

Tel.: 82-31-787-7374, Fax: 82-31-787-4057, E-mail: twkim7@snu.ac.kr

Supplementary information file includes one Table (Table S1) and two videos (Video S1-S2).

Supplementary Table S1. Correlation between the variable parameters.

*p** / *P**	Age	DM	IOP	SE	CCT	AXL	MD	PSD	HCDR	VCDR	cpRNFLT	LCD†	LCCI†
**Age**		**0.232**	-0.177	**0.483**	-0.040	**-0.390**	0.130	-0.024	-0.091	-0.085	-0.028	**-0.281**	-0.103
**DM**	**0.031**		0.007	**0.237**	0.002	-0.132	-0.061	0.028	-0.016	0.042	0.108	0.121	0.077
**IOP**	0.100	0.949		-0.207	0.196	-0.090	-0.187	0.023	0.126	0.035	-0.198	0.138	**0.213**
**SE**	**< 0.001**	**0.027**	0.054		-0.176	**-0.606**	-0.142	-0.181	-0.145	-0.008	0.088	-0.178	-0.202
**CCT**	0.728	0.986	0.084	0.120		0.179	-0.022	0.234	0.125	0.072	-0.028	-0.067	0.106
**AXL**	**< 0.001**	0.265	0.450	**< 0.001**	0.138		0.007	0.163	0.109	0.140	-0.153	0.017	0.083
**MD**	0.335	0.653	0.164	0.291	0.875	0.960		-0.132	0.166	0.150	-0.013	-0.061	-0.001
**PSD**	0.860	0.834	0.865	0.197	0.096	0.257	0.327		-0.238	-0.159	-0.033	**-0.266**	-0.156
**HCDR**	0.400	0.883	0.243	0.181	0.271	0.358	0.216	0.074		**0.740**	-0.135	**0.452**	0.202
**VCDR**	0.436	0.698	0.746	0.941	0.530	0.238	0.266	0.237	**< 0.001**		-0.154	**0.402**	**0.291**
**cpRNFLT**	0.798	0.320	0.066	0.420	0.809	0.197	0.926	0.810	0.212	0.154		0.064	-0.064
**LCD†**	**0.008**	0.265	0.202	0.099	0.559	0.886	0.651	**0.046**	**< 0.001**	**< 0.001**	0.557		**0.655**
**LCCI†**	0.342	0.480	**0.048**	0.061	0.350	0.483	0.994	0.246	**< 0.061**	**0.006**	0.557	**< 0.001**	

DM = diabetes mellitus; IOP = baseline intraocular pressure; SE = spherical equivalent; CCT = central corneal thickness; AXL = axial length; MD = mean deviation on visual field test; PSD = pattern standard deviation on visual field test; HCDR = horizontal cup-to-disc ratio; VCDR = vertical cup-to-disc ratio; cpRNFLT = circumpapillary retinal nerve fiber layer thickness; LCD = lamina cribrosa depth; LCCI = lamina cribrosa curve index.

†The mean values of the measurements from the 7 planes (from superior to inferior peripheral plane).

* Correlation coefficient (*p*) and *P* values for each pair of parameters are calculated using Pearson's correlation analysis.

Supplementary Video legends

Supplementary Video S1

Radial scans of the optic nerve head showing the W-shaped configuration of the anterior LC surface in vertical and oblique scans.

Supplementary Video S2

Raster scans of the optic nerve head showing the relatively regular configuration in the horizontal plane with a flat or U shaped appearance with differing regional steepness.
